# Crystal structure and Hirshfeld surface analysis of (*E*)-4-amino-*N*′-[1-(4-methyl­phen­yl)ethyl­idene]benzohydrazide

**DOI:** 10.1107/S205698901700857X

**Published:** 2017-06-13

**Authors:** Palaniyappan Sivajeyanthi, Muthaiah Jeevaraj, Bellarmin Edison, Kasthuri Balasubramani

**Affiliations:** a Department of Chemistry, Government Arts College (Autonomous), Thanthonimalai, Karur 639 005, Tamil Nadu, India

**Keywords:** crystal structure, Schiff base, substituted benzohydrazide, hydrogen bonding, Hirshfeld surface analysis

## Abstract

The structure of the title Schiff base, C_16_H_17_N_3_O, displays a *trans* configuration with respect to the C=N double bond, with a dihedral angle of 14.98 (9)° between the benzene rings. In the crystal, mol­ecules are linked by N—H⋯O and C—H⋯O hydrogen-bonding inter­actions, giving sheets extending across the (001) plane. Hirshfeld surface analysis gave fingerprint plots showing enrichment ratios for H⋯H, O⋯H, N⋯H and C⋯H contacts compared to C⋯C, N⋯N and C⋯N contacts, indicating a high propensity for H⋯H interactions to form in the crystal.

## Abstract 2

The title substituted benzohydrazide Schiff base compound is essentially planar, with a *trans* configuration between the benzene ring mol­ecular components, while the Hirshfeld surface analysis has been used to examine the mol­ecular inter­actions within the hydrogen-bonded structure

## Chemical context   

Schiff bases are an important class of compounds in the medicinal and pharmaceutical fields and have played a role in the development of coordination chemistry as they readily form stable complexes with most transition metals. These complexes show inter­esting properties, *e.g*. their ability to reversibly bind oxygen, catalytic activity in the hydrogenation of olefins and transfer of an amino group, photochromic properties, and complexation ability towards toxic metals (Karthikeyan *et al.*, 2006 ▸; Khattab *et al.*, 2005 ▸; Küçükgüzel *et al.*, 2006 ▸). Hydrazone Schiff base compounds (Cao *et al.*, 2009 ▸; Zhou & Yang, 2010 ▸; Zhang *et al.*, 2009 ▸), derived from the reaction of aldehydes with hydrazines have been shown to possess excellent biological activities, such as anti-bacterial, anti-convulsant and anti-tubercular (Bernhardt *et al.*, 2005 ▸; Armstrong *et al.*, 2003 ▸). As part of our studies in this area, the title Schiff base compound (*E*)-4-amino-*N*′-(1-(*p*-tol­yl)ethyl­idene)benzo­hydrazide, was prepared and the crystal structure is reported herein. Hirshfeld surface analysis was also performed for visualizing and qu­anti­fying inter­molecular inter­actions in the crystal packing of the compound.
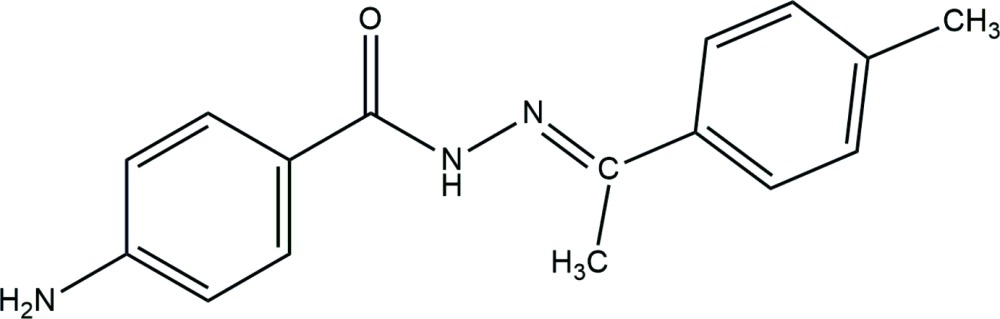


## Structural commentary   

**Figure 1:**
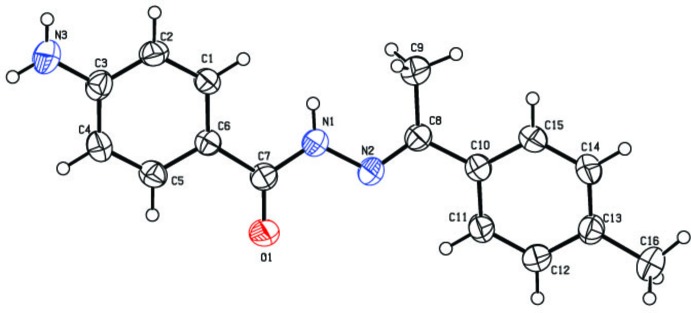
The mol­ecular structure of the title compound, with displacement ellipsoids drawn at the 50% probability level.

The asymmetric unit of the title compound contains one independent mol­ecule (Fig. 1 ▸), displaying a *trans* conformation with respect to its C=N double bond. The dihedral angle between the benzene rings is 14.98 (9)°. All the bond lengths are within normal ranges. The C8=N2 and C7=O1 bond lengths [1.281 (2) and 1.231 (2) Å, respectively] confirm their double-bond character, whereas the C3—N3, C7—N1 and N1—N2 values [1.365 (3), 1.357 (2) and 1.388 (2) Å, respectively]; these C—N bonds are much shorter than (nominal) isolated C—N bonds (1.46 Å) due to conjugation.

## Supra­molecular features   

**Figure 2:**
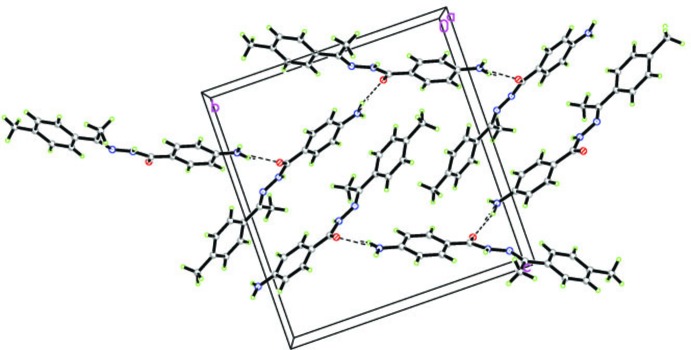
Crystal packing of the title compound in the unit cell, showing mol­ecules linked across *b via* N—H⋯O hydrogen bonds (dashed lines).

**Figure 3:**
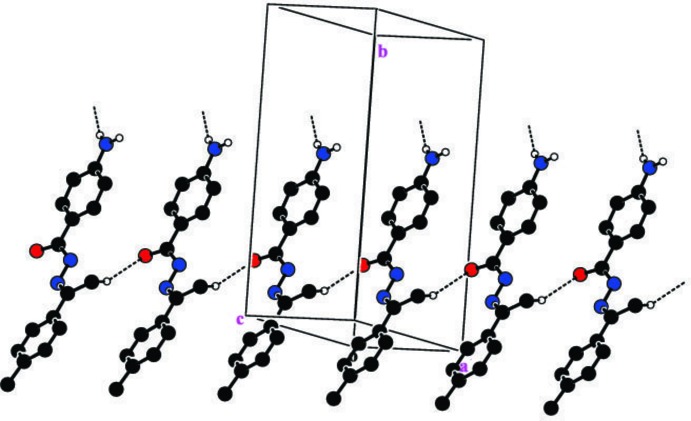
The crystal packing in the title compound in which mol­ecules are linked across *a via* weak C—H⋯O hydrogen bonds (dashed lines). H atoms not involved in hydrogen-bonding inter­actions have been omitted.

In the crystal, two types of inter­molecular hydrogen-bonding inter­actions are present (Table 1 ▸). The N3—H1*N*3⋯O1^i^ hydrogen bond between the amino group and a symmetry-related carbonyl group generates zigzag chains extending along the *b-*axis direction, as shown in Fig. 2 ▸. The secondary weak methyl C9—H9*A*⋯O1^ii^ hydrogen-bonding inter­actions extend the structure across *a* (Fig. 3 ▸), generating a layer lying parallel to (001). No reasonable acceptors could be identified for either the second amine N3 H atom or the hydrazide N1 H atom.

## Hirshfeld surface analysis   

**Figure 4:**
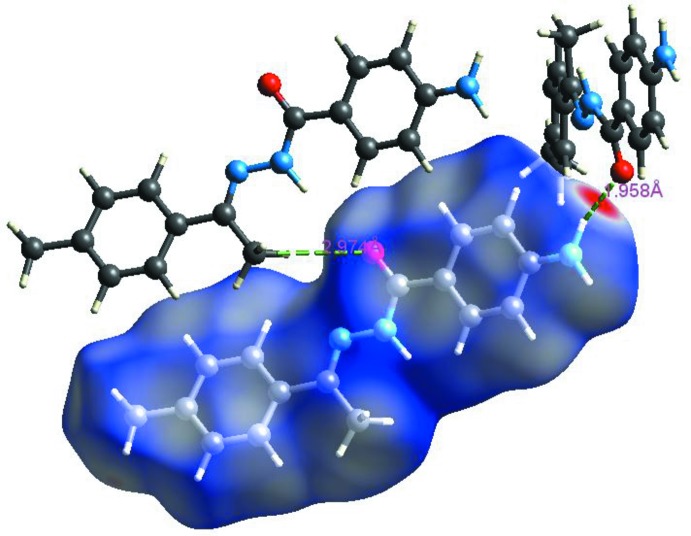
Hirshfeld surfaces mapped over *d*_norm_ for the title compound.

Hirshfeld surfaces and their associated two-dimensional fingerprint plots (Soman *et al.*, 2014 ▸) have been used to qu­antify the various inter­molecular inter­actions in the title compound. The Hirshfeld surface of a mol­ecule is mapped using the descriptor *d*_norm_ which encompasses two factors: one is *d*_e_, representing the distance of any surface point nearest to the inter­nal atoms, and the other one is *d*_i_, representing the distance of the surface point nearest to the exterior atoms and also with the van der Waals radii of the atoms (Dalal *et al.*, 2015 ▸). The Hirshfeld surfaces mapped over *d*_norm_ (range of −0.502–1.427 Å) are displayed in Fig. 4 ▸. The surfaces are shown as transparent to allow visualization of the mol­ecule. The dominant inter­action between oxygen (O) and hydrogen (H) atoms can be observed in the Hirshfeld surface as the red areas (Fig. 4 ▸). Other visible spots in the Hirshfeld surfaces correspond to C—H and H—H contacts.

**Figure 5:**
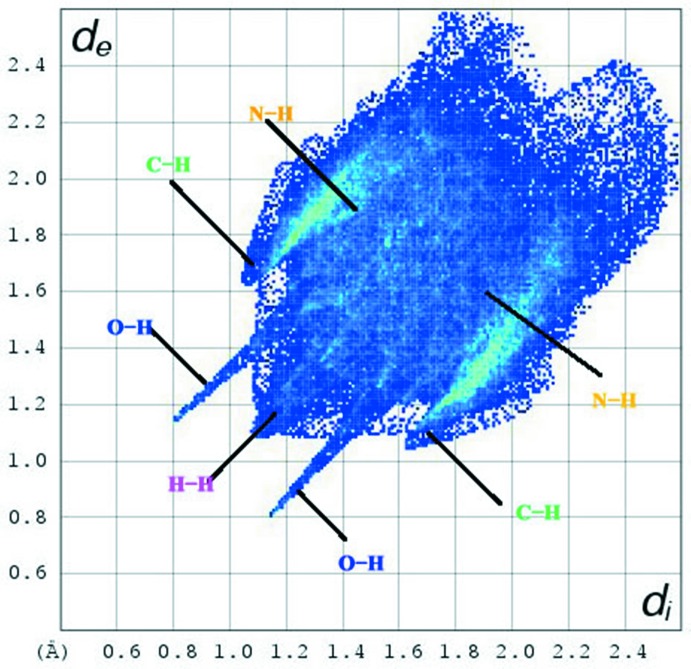
Two-dimensional fingerprint plots of the title compound.

**Figure 6:**
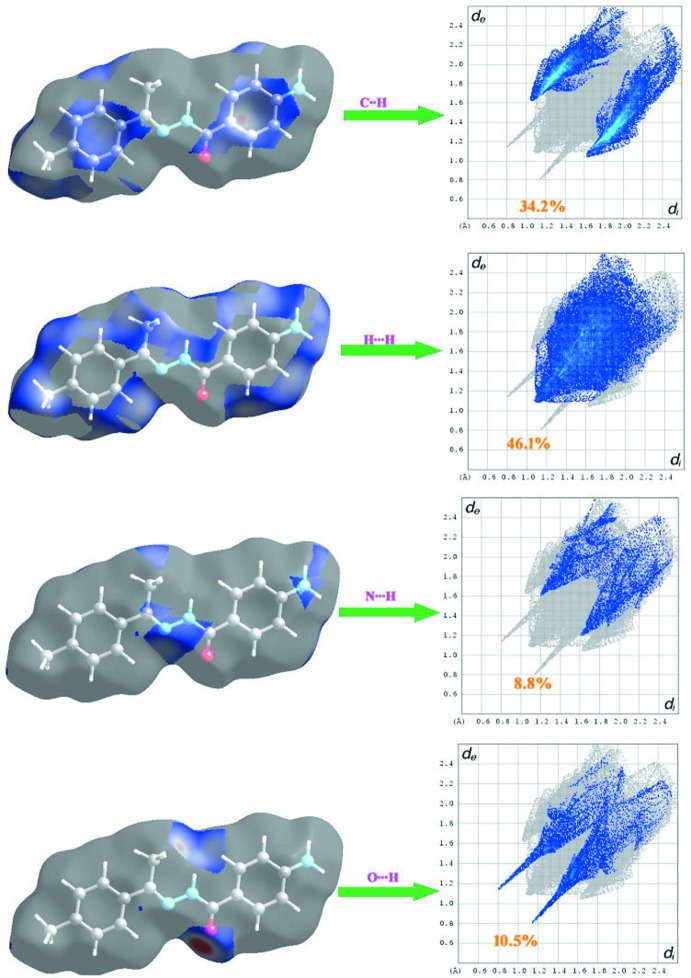
Two-dimensional fingerprint plots with a *d*_norm_ view of the C⋯H/H⋯C (34.2%), H⋯H (46.1%), N⋯H/H⋯N (8.8%) and O⋯H/H⋯O (10.5%) contacts in the title compound.

The inter­molecular inter­actions of the title compound are shown in the 2D fingerprint plots shown in Fig. 5 ▸. H⋯H (46.1%) contacts make the largest contribution to the Hirshfeld surfaces. O⋯H/H⋯O (10.5%), inter­actions are represented by left-side blue spikes, top and bottom. The pale yellow N⋯H/H⋯N (8.8%) inter­actions are near the C⋯H regions while the green C⋯H/H⋯C inter­actions (34.2%) are between the N—H and O—H regions. The whole fingerprint region and all other inter­actions, which are a combination of *d*_e_ and *d*_i_, are displayed in Fig. 6 ▸.

## Synthesis and crystallization   

The title compound was synthesized by the reaction of a 1:1 molar ratio mixture of a hot methano­lic solution (20 mL) of 4-amini­benzoic­hydrazide (0.151 mg, Aldrich) and a hot methano­lic solution of 4-methyl­aceto­phenone (0.134 mg, Aldrich), which was refluxed for 8 h. The solution was then cooled and kept at room temperature after which colourless block-shaped crystals suitable for the X-ray analysis were obtained in a few days.

## Refinement   

Crystal data, data collection and structure refinement details are summarized in Table 2 ▸. Hydrogen atoms were positioned geometrically (N—H = 0.86 Å, and C—H = 0.93 or 0.96 Å) and were refined using a riding model, with *U*_iso_(H) = 1.2 *U*_eq_(N, C) or 1.5*U*_eq_(methyl C). One reflection (011) was considered to be affected by the beamstop.

## Supplementary Material

Crystal structure: contains datablock(s) global, I. DOI: 10.1107/S205698901700857X/zs2381sup1.cif

Structure factors: contains datablock(s) I. DOI: 10.1107/S205698901700857X/zs2381Isup2.hkl

Supporting information file. DOI: 10.1107/S205698901700857X/zs2381Isup3.cml

CCDC reference: 1554995

Additional supporting information:  crystallographic information; 3D view; checkCIF report

## Figures and Tables

**Table 1:** Hydrogen-bond geometry (Å, °)

*D*—H⋯*A*	*D*—H	H⋯*A*	*D*⋯*A*	*D*—H⋯*A*
N3—H1*N*3⋯O1^i^	0.86	2.10	2.914 (2)	159
C9—H9*A*⋯O1^ii^	0.96	2.60	3.475 (3)	152

Symmetry codes: (i) 
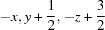
; (ii) 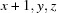.

**Table 2:** Experimental details

Crystal data	
Chemical formula	C_16_H_17_N_3_O
*M*_r_	267.33
Crystal system, space group	Orthorhombic, *P*2_1_2_1_2_1_
Temperature (K)	296
*a*, *b*, *c* (Å)	5.7011 (4), 15.4836 (10), 16.2128 (10)
*V* (Å^3^)	1431.16 (16)
*Z*	4
Radiation type	Mo *K*α
μ (mm^−1^)	0.08
Crystal size (mm)	0.30 × 0.20 × 0.20

Data collection	
Diffractometer	Bruker Kappa APEXII CCD
Absorption correction	Multi-scan (*SADABS*; Bruker, 2004 ▸)
*T*_min_, *T*_max_	0.976, 0.984
No. of measured, independent and observed [*I* > 2σ(*I*)] reflections	17427, 3501, 2784
*R*_int_	0.027
(sin θ/λ)_max_ (Å^−1^)	0.666

Refinement	
*R*[*F* ^2^ > 2σ(*F* ^2^)], *wR*(*F* ^2^), *S*	0.045, 0.141, 1.05
No. of reflections	3501
No. of parameters	182
H-atom treatment	H-atom parameters constrained
Δρ_max_, Δρ_min_ (e Å^−3^)	0.17, −0.20
Absolute structure	Flack (1983 ▸), 1489 Friedel pairs
Absolute structure parameter	0.6 (19)

Computer programs: *APEX2* (Bruker, 2004 ▸), *APEX2*, *SAINT* and *XPREP* (Bruker, 2004 ▸), *SIR92* (Altomare *et al.*, 1993 ▸), *SHELXL97* (Sheldrick, 2008 ▸), *ORTEP-3 for Windows* (Farrugia, 2012 ▸) and *Mercury* (Macrae *et al.*, 2008 ▸).

## supplementary crystallographic information

### Crystal data

**Table:**

C_16_H_17_N_3_O	*F*(000) = 568
*M**_r_* = 267.33	*D*_x_ = 1.241 Mg m^−^^3^
Orthorhombic, *P*2_1_2_1_2_1_	Mo *K*α radiation, λ = 0.71073 Å
Hall symbol: P 2ac 2ab	Cell parameters from 6816 reflections
*a* = 5.7011 (4) Å	θ = 5.0–49.0°
*b* = 15.4836 (10) Å	µ = 0.08 mm^−^^1^
*c* = 16.2128 (10) Å	*T* = 296 K
*V* = 1431.16 (16) Å^3^	Block, colorless
*Z* = 4	0.30 × 0.20 × 0.20 mm

### Data collection

**Table:**

Bruker Kappa APEXII CCD diffractometer	3501 independent reflections
Radiation source: fine-focus sealed tube	2784 reflections with *I* > 2σ(*I*)
Graphite monochromator	*R*_int_ = 0.027
ω and φ scan	θ_max_ = 28.3°, θ_min_ = 1.8°
Absorption correction: multi-scan (SADABS; Bruker, 2004)	*h* = −7→7
*T*_min_ = 0.976, *T*_max_ = 0.984	*k* = −17→20
17427 measured reflections	*l* = −21→21

### Refinement

**Table:**

Refinement on *F*^2^	Secondary atom site location: difference Fourier map
Least-squares matrix: full	Hydrogen site location: inferred from neighbouring sites
*R*[*F*^2^ > 2σ(*F*^2^)] = 0.045	H-atom parameters constrained
*wR*(*F*^2^) = 0.141	*w* = 1/[σ^2^(*F*_o_^2^) + (0.0847*P*)^2^ + 0.1161*P*] where *P* = (*F*_o_^2^ + 2*F*_c_^2^)/3
*S* = 1.05	(Δ/σ)_max_ < 0.001
3501 reflections	Δρ_max_ = 0.17 e Å^−^^3^
182 parameters	Δρ_min_ = −0.20 e Å^−^^3^
0 restraints	Absolute structure: Flack (1983), 1489 Friedel pairs
Primary atom site location: structure-invariant direct methods	Absolute structure parameter: 0.6 (19)

### Special details

**Table:**

Geometry. All esds (except the esd in the dihedral angle between two l.s. planes) are estimated using the full covariance matrix. The cell esds are taken into account individually in the estimation of esds in distances, angles and torsion angles; correlations between esds in cell parameters are only used when they are defined by crystal symmetry. An approximate (isotropic) treatment of cell esds is used for estimating esds involving l.s. planes.
Refinement. Refinement of F^2^ against ALL reflections. The weighted R-factor wR and goodness of fit S are based on F^2^, conventional R-factors R are based on F, with F set to zero for negative F^2^. The threshold expression of F^2^ > 2sigma(F^2^) is used only for calculating R-factors(gt) etc. and is not relevant to the choice of reflections for refinement. R-factors based on F^2^ are statistically about twice as large as those based on F, and R- factors based on ALL data will be even larger.

### Fractional atomic coordinates and isotropic or equivalent isotropic displacement parameters (Å^2^)

**Table:**

	*x*	*y*	*z*	*U*_iso_*/*U*_eq_	
N1	0.2405 (3)	0.16299 (10)	0.88410 (10)	0.0543 (4)	
H1N	0.3848	0.1789	0.8871	0.065*	
N2	0.1715 (3)	0.08479 (10)	0.91817 (10)	0.0519 (4)	
C11	0.0364 (4)	−0.07382 (12)	0.98126 (12)	0.0518 (5)	
H11	−0.0509	−0.0504	0.9383	0.062*	
O1	−0.1236 (3)	0.19158 (9)	0.83572 (11)	0.0718 (5)	
C5	0.0187 (3)	0.35074 (11)	0.77149 (11)	0.0477 (4)	
H5	−0.1250	0.3283	0.7548	0.057*	
C8	0.3188 (3)	0.04879 (11)	0.96690 (11)	0.0450 (4)	
C10	0.2444 (3)	−0.03365 (11)	1.00549 (10)	0.0434 (4)	
C12	−0.0420 (4)	−0.14777 (12)	1.02002 (12)	0.0557 (5)	
H12	−0.1814	−0.1730	1.0026	0.067*	
N3	0.3577 (4)	0.55025 (11)	0.74979 (15)	0.0824 (6)	
H2N3	0.4905	0.5709	0.7654	0.099*	
H1N3	0.2650	0.5811	0.7201	0.099*	
C3	0.2940 (4)	0.46854 (11)	0.77215 (12)	0.0514 (4)	
C4	0.0803 (4)	0.43309 (12)	0.74768 (11)	0.0518 (4)	
H4	−0.0215	0.4653	0.7151	0.062*	
C6	0.1661 (3)	0.30033 (10)	0.81983 (10)	0.0408 (4)	
C7	0.0810 (3)	0.21446 (11)	0.84607 (11)	0.0472 (4)	
C13	0.0821 (4)	−0.18536 (12)	1.08434 (11)	0.0509 (4)	
C16	−0.0112 (5)	−0.26470 (15)	1.12687 (15)	0.0721 (7)	
H16A	0.0964	−0.2823	1.1692	0.108*	
H16B	−0.0287	−0.3105	1.0874	0.108*	
H16C	−0.1608	−0.2519	1.1511	0.108*	
C2	0.4428 (3)	0.41823 (12)	0.82025 (13)	0.0527 (4)	
H2	0.5868	0.4405	0.8368	0.063*	
C1	0.3806 (3)	0.33609 (11)	0.84366 (12)	0.0474 (4)	
H1	0.4829	0.3038	0.8759	0.057*	
C9	0.5540 (4)	0.08701 (17)	0.98774 (16)	0.0755 (7)	
H9A	0.6427	0.0952	0.9380	0.113*	
H9B	0.6372	0.0486	1.0238	0.113*	
H9C	0.5322	0.1416	1.0147	0.113*	
C15	0.3706 (4)	−0.07226 (13)	1.06905 (11)	0.0529 (5)	
H15	0.5109	−0.0475	1.0863	0.063*	
C14	0.2906 (4)	−0.14736 (14)	1.10736 (13)	0.0574 (5)	
H14	0.3796	−0.1722	1.1492	0.069*	

### Atomic displacement parameters (Å^2^)

**Table:**

	*U*^11^	*U*^22^	*U*^33^	*U*^12^	*U*^13^	*U*^23^
N1	0.0503 (8)	0.0419 (7)	0.0706 (10)	−0.0063 (7)	−0.0069 (8)	0.0095 (7)
N2	0.0532 (9)	0.0386 (7)	0.0638 (9)	−0.0062 (7)	−0.0056 (7)	0.0055 (7)
C11	0.0551 (11)	0.0474 (9)	0.0529 (10)	−0.0027 (9)	−0.0118 (9)	0.0072 (8)
O1	0.0552 (9)	0.0525 (8)	0.1078 (12)	−0.0153 (7)	−0.0246 (9)	0.0154 (8)
C5	0.0450 (9)	0.0491 (9)	0.0489 (9)	0.0012 (8)	−0.0064 (8)	−0.0028 (8)
C8	0.0472 (9)	0.0423 (8)	0.0455 (8)	−0.0025 (7)	0.0007 (8)	−0.0019 (7)
C10	0.0446 (9)	0.0428 (8)	0.0429 (8)	0.0027 (8)	−0.0001 (7)	−0.0014 (7)
C12	0.0513 (11)	0.0527 (10)	0.0632 (12)	−0.0089 (9)	−0.0074 (10)	0.0053 (9)
N3	0.0756 (13)	0.0518 (10)	0.1198 (17)	−0.0061 (10)	−0.0042 (13)	0.0306 (11)
C3	0.0549 (11)	0.0407 (9)	0.0585 (10)	0.0024 (8)	0.0096 (9)	0.0037 (8)
C4	0.0553 (11)	0.0483 (9)	0.0518 (10)	0.0099 (8)	−0.0029 (9)	0.0057 (8)
C6	0.0417 (8)	0.0383 (7)	0.0424 (8)	−0.0001 (7)	−0.0012 (7)	−0.0031 (6)
C7	0.0500 (10)	0.0391 (8)	0.0527 (9)	−0.0048 (8)	−0.0078 (8)	−0.0021 (7)
C13	0.0527 (11)	0.0493 (9)	0.0507 (10)	0.0021 (9)	0.0060 (8)	0.0079 (8)
C16	0.0745 (15)	0.0686 (13)	0.0731 (14)	−0.0103 (12)	0.0057 (12)	0.0248 (11)
C2	0.0418 (10)	0.0453 (9)	0.0709 (11)	−0.0054 (8)	−0.0022 (9)	0.0010 (8)
C1	0.0417 (9)	0.0418 (8)	0.0586 (10)	0.0006 (8)	−0.0083 (8)	0.0035 (7)
C9	0.0624 (14)	0.0771 (14)	0.0870 (15)	−0.0209 (13)	−0.0199 (12)	0.0280 (12)
C15	0.0447 (10)	0.0606 (11)	0.0534 (10)	−0.0010 (9)	−0.0079 (8)	0.0058 (9)
C14	0.0545 (12)	0.0640 (12)	0.0536 (10)	0.0040 (10)	−0.0068 (9)	0.0161 (9)

### Geometric parameters (Å, º)

**Table:**

N1—C7	1.357 (2)	C3—C2	1.391 (3)
N1—N2	1.388 (2)	C3—C4	1.394 (3)
N1—H1N	0.8600	C4—H4	0.9300
N2—C8	1.281 (2)	C6—C1	1.397 (2)
C11—C12	1.380 (3)	C6—C7	1.478 (2)
C11—C10	1.395 (3)	C13—C14	1.378 (3)
C11—H11	0.9300	C13—C16	1.506 (3)
O1—C7	1.231 (2)	C16—H16A	0.9600
C5—C4	1.378 (2)	C16—H16B	0.9600
C5—C6	1.389 (2)	C16—H16C	0.9600
C5—H5	0.9300	C2—C1	1.374 (3)
C8—C10	1.483 (2)	C2—H2	0.9300
C8—C9	1.504 (3)	C1—H1	0.9300
C10—C15	1.392 (3)	C9—H9A	0.9600
C12—C13	1.388 (3)	C9—H9B	0.9600
C12—H12	0.9300	C9—H9C	0.9600
N3—C3	1.365 (2)	C15—C14	1.395 (3)
N3—H2N3	0.8600	C15—H15	0.9300
N3—H1N3	0.8600	C14—H14	0.9300
			
C7—N1—N2	120.20 (17)	O1—C7—N1	121.87 (17)
C7—N1—H1N	119.9	O1—C7—C6	122.06 (17)
N2—N1—H1N	119.9	N1—C7—C6	116.05 (16)
C8—N2—N1	116.05 (16)	C14—C13—C12	117.64 (17)
C12—C11—C10	121.11 (18)	C14—C13—C16	121.95 (18)
C12—C11—H11	119.4	C12—C13—C16	120.41 (19)
C10—C11—H11	119.4	C13—C16—H16A	109.5
C4—C5—C6	121.61 (18)	C13—C16—H16B	109.5
C4—C5—H5	119.2	H16A—C16—H16B	109.5
C6—C5—H5	119.2	C13—C16—H16C	109.5
N2—C8—C10	116.57 (16)	H16A—C16—H16C	109.5
N2—C8—C9	123.50 (17)	H16B—C16—H16C	109.5
C10—C8—C9	119.90 (17)	C1—C2—C3	121.04 (17)
C15—C10—C11	117.15 (17)	C1—C2—H2	119.5
C15—C10—C8	122.29 (17)	C3—C2—H2	119.5
C11—C10—C8	120.51 (16)	C2—C1—C6	121.12 (17)
C11—C12—C13	121.66 (19)	C2—C1—H1	119.4
C11—C12—H12	119.2	C6—C1—H1	119.4
C13—C12—H12	119.2	C8—C9—H9A	109.5
C3—N3—H2N3	120.0	C8—C9—H9B	109.5
C3—N3—H1N3	120.0	H9A—C9—H9B	109.5
H2N3—N3—H1N3	120.0	C8—C9—H9C	109.5
N3—C3—C2	120.36 (19)	H9A—C9—H9C	109.5
N3—C3—C4	121.45 (19)	H9B—C9—H9C	109.5
C2—C3—C4	118.18 (16)	C10—C15—C14	121.25 (19)
C5—C4—C3	120.48 (18)	C10—C15—H15	119.4
C5—C4—H4	119.8	C14—C15—H15	119.4
C3—C4—H4	119.8	C13—C14—C15	121.16 (18)
C5—C6—C1	117.57 (15)	C13—C14—H14	119.4
C5—C6—C7	118.01 (16)	C15—C14—H14	119.4
C1—C6—C7	124.35 (15)		
			
C7—N1—N2—C8	−166.25 (17)	C5—C6—C7—O1	−9.6 (3)
N2—N1—C7—O1	−4.9 (3)	C5—C6—C7—N1	171.91 (16)
N2—N1—C7—C6	173.63 (15)	N2—C8—C10—C11	7.5 (3)
N1—N2—C8—C9	0.2 (3)	N2—C8—C10—C15	−169.65 (18)
N1—N2—C8—C10	178.39 (15)	C9—C8—C10—C11	−174.22 (18)
C6—C1—C2—C3	0.1 (3)	C9—C8—C10—C15	8.6 (3)
C2—C1—C6—C5	0.2 (3)	C8—C10—C11—C12	−176.11 (18)
C2—C1—C6—C7	−176.59 (17)	C15—C10—C11—C12	1.2 (3)
C1—C2—C3—N3	179.6 (2)	C8—C10—C15—C14	176.54 (18)
C1—C2—C3—C4	−0.5 (3)	C11—C10—C15—C14	−0.7 (3)
N3—C3—C4—C5	−179.5 (2)	C10—C11—C12—C13	−0.1 (3)
C2—C3—C4—C5	0.6 (3)	C11—C12—C13—C14	−1.4 (3)
C3—C4—C5—C6	−0.4 (3)	C11—C12—C13—C16	178.4 (2)
C4—C5—C6—C1	−0.1 (3)	C12—C13—C14—C15	1.9 (3)
C4—C5—C6—C7	176.93 (17)	C16—C13—C14—C15	−177.9 (2)
C1—C6—C7—O1	167.14 (18)	C13—C14—C15—C10	−0.9 (3)
C1—C6—C7—N1	−11.3 (3)		

### Hydrogen-bond geometry (Å, º)

**Table:**

*D*—H···*A*	*D*—H	H···*A*	*D*···*A*	*D*—H···*A*
N3—H1*N*3···O1^i^	0.86	2.10	2.914 (2)	159
C9—H9*A*···O1^ii^	0.96	2.60	3.475 (3)	152

Symmetry codes: (i) −*x*, *y*+1/2, −*z*+3/2; (ii) *x*+1, *y*, *z*.
